# The Characteristics of Treated Pulmonary Arterial Hypertension Patients in Ontario

**DOI:** 10.1155/2016/6279250

**Published:** 2016-04-11

**Authors:** Haris M. Vaid, Ximena Camacho, John T. Granton, Muhammad M. Mamdani, Zhan Yao, Samantha Singh, David N. Juurlink, Tara Gomes

**Affiliations:** ^1^School of Medicine, Queen's University, 15 Arch Street, Kingston, ON, Canada K7L 3N6; ^2^The Institute for Clinical Evaluative Sciences, 2075 Bayview Avenue G1 06, Toronto, ON, Canada M4N 3M5; ^3^Toronto General Hospital, 200 Elizabeth Street, Toronto, ON, Canada M5G 2C4; ^4^University Health Network, 399 Bathurst Street, Toronto, ON, Canada M5T 2S8; ^5^Department of Medicine, The University of Toronto, 1 King's College Circle No. 3172, Toronto, ON, Canada M5S 1A8; ^6^The Department of Medicine, St. Michael's Hospital, 30 Bond Street, Toronto, ON, Canada M5B 1W8; ^7^Department of Health Policy, Management, and Evaluation, The University of Toronto, 155 College Street, Toronto, ON, Canada M5T 3M6; ^8^The Leslie Dan Faculty of Pharmacy, The University of Toronto, 144 College Street, Toronto, ON, Canada M5S 3M2; ^9^Keenan Research Centre, The Li Ka Shing Knowledge Institute, St. Michael's Hospital, 209 Victoria Street, Toronto, ON, Canada M5B 1T8; ^10^The Sunnybrook Research Institute, 2075 Bayview Avenue, Toronto, ON, Canada M4N 3M5; ^11^Department of Pediatrics, The University of Toronto, 555 University Avenue Black Wing, 1436 Toronto, ON, Canada M5G 1X8

## Abstract

*Background*. There are no Canadian prevalence studies on pulmonary arterial hypertension (PAH) to date. We described the characteristics of treated PAH patients and the healthcare utilization and costs associated with PAH in a population of public drug plan beneficiaries in Ontario, Canada.* Methods*. A retrospective cross-sectional analysis was conducted between April 2010 and March 2011 to identify treated PAH patients using population-based health administrative databases. We investigated demographic and clinical characteristics of treated PAH patients and conducted a cohort study to determine treatment patterns, healthcare utilization, and associated costs, over a one-year follow-up period (March 2012).* Results*. We identified 326 treated PAH cases in Ontario's publicly funded drug plan. Overall mean age was 59.4 years (±20.3 years) and over 77% of cases were women (*n* = 251). Combination therapy was used to treat 22.9% (*n* = 69) of cases, costing an average of $4,569 (SD $1,544) per month. Median monthly healthcare costs were $264 (IQR $96–$747) for those who survived and $2,021 (IQR $993–$6,399) for those who died over a one-year period, respectively (*p* < 0.01).* Conclusions*. PAH care in Ontario is complex and has high healthcare costs. This data may help guide towards improved patient management.

## 1. Introduction

Pulmonary arterial hypertension (PAH) is a rare condition, which is often unrecognized, resulting in delayed diagnosis and treatment [1]. PAH is associated with substantial morbidity and mortality, resulting in significant health and economic impact on patients and the healthcare system. PAH has a very poor prognosis if left untreated, with an average survival time of 2.8 years after diagnosis [2]. Although there are several PAH registries worldwide [3–13], PAH is understudied in Canada. There are only a few Canadian epidemiological studies in select subpopulations [14, 15], and to the best of our knowledge, there are currently no Canadian disease registries or prevalence estimates on PAH using population-based data.

Knowledge on the economic burden associated with PAH in Canada is also lacking, although a few Canadian cost analyses using a model-based approach comparing specific PAH treatments have previously been attempted [16–18]. Elsewhere, retrospective analyses revealed PAH-associated healthcare costs averaged $4,236 per patient per month in the United States [19] and €47,400 per patient per year in Germany [20]. In both studies, costs were predominantly driven by PAH medication.

The costs associated with PAH management are high in Canada. Although there is no cure, targeted therapies may help to manage associated symptoms and improve survival [21]. The three classes of PAH drugs approved in Canada include prostacyclin (PRO), endothelin receptor antagonists (ERA), and phosphodiesterase-5 inhibitors (PDE5is). Riociguat is the first drug in a novel class of soluble guanylate cyclase inhibitors, which is newly approved in Canada. These therapies vary by routes of administration, costs, and side effects [22]. PDE5i and ERA costs have previously been reported to range between $10,000 and $40,000 per person per year and intravenous and subcutaneous therapies, such as PROs, range between $80,000 and $100,000 per person per year [23]. When response to monotherapy is inadequate, combination therapy is endorsed by several societies, pulmonary hypertension treatment guidelines, and expert consensus [23–27]. High costs have nevertheless made combination therapy tightly controlled by government and private payers, limiting its widespread use.

Using large health registry data, we sought to describe the characteristics of treated PAH patients in a population of individuals eligible for government-funded drug coverage in Ontario and examine their annual health services utilization and costs.

## 2. Methods

### 2.1. Study Design

We conducted a cross-sectional analysis to examine the clinical characteristics of patients who received a prescription for a PAH medication and who were eligible for public drug coverage in Ontario, between April 1, 2010, and March 31, 2011. We also conducted a cohort study among these identified PAH medication users, between April 1, 2011, and March 31, 2012, to examine medication and resource utilization patterns, costs, and one-year mortality rate. In Ontario, the government provides prescription drug coverage for residents who qualify for social assistance, live in a long-term care facility, receive home care, have high prescription drug costs relative to their household income, or are aged 65 years and older. This project was approved by the Research Ethics Board of Sunnybrook Health Sciences Centre, Toronto, Canada.

### 2.2. Data Sources

We used the Ontario Drug Benefit (ODB) Program database, which contains prescription dispensation data for all Ontarians eligible for publically funded drug coverage in Ontario, to identify public drug plan eligibility, medication use, and their costs (including PAH drugs). We used the Canadian Institute for Health Information Discharge Abstract Database (CIHI-DAD) to identify hospital admissions and their associated costs and the CIHI National Ambulatory Care Reporting System (CIHI-NACRS) to identify emergency department visits and their associated costs. We also used CIHI-DAD and CIHI-NACRS to identify patient comorbidities using the International Classification of Diseases, 10th Revision (ICD-10) codes. We obtained physician-billing data from the Ontario Health Insurance Plan database, and we retrieved demographic information and dates of death, using the Ontario Registered Persons Database, which contains information for anyone who has ever received an Ontario health card number. All patients were anonymously identified and linked using an encrypted 10-digit health card number.

### 2.3. Cohort Definition

We identified a cohort of treated PAH patients, defined as those who filled at least one prescription for an ERA (bosentan or ambrisentan), PDE5i (sildenafil or tadalafil), or PRO (epoprostenol or treprostinil), between April 1, 2010, and March 31, 2011, and who were alive as of April 1, 2011. We did not include riociguat in our analysis because it was not available on the public drug formulary over the study period.

### 2.4. Outcome Definitions

#### 2.4.1. PAH Drug Utilization and Costs

We followed all treated PAH patients who were alive on April 1, 2011, forward for one year (to March 31, 2012), to define drug treatment and health services utilization patterns. We defined “single therapy users” as those who were prescribed a single PAH drug type (ERA, PDE5i, or PRO) over the follow-up period and “combination therapy users” as those who were prescribed two or more PAH drug types, where the interval of use of the first and subsequent drugs overlapped. We also calculated the total cost of drugs per individual for single and combination therapy users over the study period using associated billing data.

#### 2.4.2. Mortality

We defined the one-year mortality rate as the proportion of all treated PAH patients who died of any cause during the one-year follow-up period. Those who died during this period were assigned to the* Deceased Cohort*, and those that were alive as of March 31, 2012, were assigned to the* Survivor Cohort*. We assessed these cohorts separately for health services utilization and associated costs.

#### 2.4.3. Health Services Utilization and Costs

We assessed annual PAH drug and annual health services utilization patterns using physician visits, hospitalizations, and emergency department visits, from April 1, 2011, to March 31, 2012. We also assessed the associated standardized monthly costs for these health services. We restricted the designation of physician visits to one claim per person, per physician, daily, and to nonlaboratory claims occurring in outpatient settings. We defined hospitalizations as acute inpatient admissions and same-day surgeries. We assessed person level costs in accordance with the Health System Performance Research Network's guidelines, described in detail elsewhere [28].

### 2.5. Statistical Analyses

We reported patient demographics and clinical characteristics using categorical or binary variables; age was reported with mean and standard deviations. For baseline characteristics, we compared differences in proportions between age groups (<65 versus ≥65) using chi-square analysis. We also reported the proportion of individuals that used specific health services, or PAH drugs, with categorical or binary variables, and compared proportions between age groups, or between Survival and Deceased Cohorts, using chi-square analysis. We reported health services utilization frequency with median and interquartile range and compared metrics between age groups, or between cohorts, using the Kruskal-Wallis test. We reported all cost metrics for health services utilization and PAH drugs with median and interquartile range, or mean and standard deviation, and compared them between age groups, or between cohorts, using the Kruskal-Wallis test or one-way ANOVA, respectively. All analyses were performed using SAS software (version 9.2, SAS Institute Inc.) and STATA (version 13, Stata Corp, College Station, Texas) and used a type I error rate of 0.05 as the threshold for statistical significance.

## 3. Results

### 3.1. Clinical Characteristics of Treated PAH Patients

Among the 2,623,692 individuals who were eligible for public drug coverage in Ontario (926,344 of age < 65 and 1,697,348 of age ≥ 65), we identified 326 who were treated for PAH and alive as of April 1, 2011. The overall mean age was 59.4 years (±20.3 years) and over three-quarters of treated PAH patients were women (*n* = 251; 77%). Overall, chronic obstructive pulmonary disease (*n* = 147; 45.1%) was the most commonly reported comorbidity. Patient comorbidities differed significantly between age groups for several conditions including respiratory diseases (*n* = 98  (60.9%) of 161 and *n* = 56  (33.9%) of 165, *p* < 0.0001 for age ≥ 65 and age < 65, resp.) and diabetes (*n* = 52  (32.3%) of 161 and *n* = 27  (16.4%) of 165, *p* < 0.01, for age ≥ 65 and age < 65, resp.). Overall, 44.5% of individuals were on antihypertensive medications (Table 1).

### 3.2. PAH Drug Utilization and Costs

Most individuals were treated with single PAH drug therapy over the 1-year follow-up (*n* = 232  (77.1%) of 301), and ERAs were the most commonly prescribed type of single therapy medication (*n* = 140  (60.3%) of 232) (Table 2). Dual PDE5i plus ERA was the most frequently prescribed form of combination therapy (*n* = 62  (89.9%) of 69).

Among single therapy users, the average drug costs were $2,801 (SD $1,550) per month and did not significantly differ by age group ($2,833 (SD $1,546) and $2,767 (SD $1,561) for age < 65 and age ≥ 65, resp.; *p* = 0.746) (see E-Appendix 1 in Supplementary Material available online at http://dx.doi.org/10.1155/2016/6279250). The average costs for single PDE5, ERA, and PROs therapy were $880 (SD $380), $3,609 (SD $1,043), and $3,324 (SD $1,451) per month, respectively.

Compared to single therapy, the average drug costs for combination therapy were significantly higher ($4,569 (SD $1,544) per month for combination therapy versus $2,801 (SD $1,550) for single therapy; *p* < 0.01). For combination therapy users, the average monthly drug costs also did not differ by age group: ($4,420 (SD $1,504) and $4,713 (SD $1,591) for age < 65 and age ≥ 65, resp.; *p* = 0.435).

### 3.3. Mortality

The overall one-year mortality rate among treated PAH patients was 10.4% (*N* = 34). This rate differed significantly by age group (age ≥ 65: 14.9% versus age < 65: 6.1%; *p* < 0.01).

### 3.4. Health Services Utilization and Costs

Overall, 49.3% (*n* = 144 of 292) of the Survivor Cohort had at least one hospitalization over the one-year follow-up (Table 3). This differed significantly by age, with the older cohort (age ≥ 65) having a higher proportion of hospitalization than the younger (age < 65) cohort (59.1% versus 40.6%; *p* = 0.002). The overall median health services costs were significantly higher in the Deceased Cohort ($2,021 (IQR $993–$6,399) per month) compared to the Survivor Cohort ($264 (IQR $96–$747) per month; *p* < 0.01). This was largely influenced by higher hospitalization costs in the time between start of follow-up and death (median $1,820 (IQR $767–$6,332) per month for those patients who died over the follow-up period compared to $405 (IQR $152–$889) per month for those who survived for 1 year; *p* < 0.01). Compared to the Survivor Cohort, those in the Deceased Cohort also had a higher proportion of hospitalization (*p* < 0.01) and emergency department visits (*p* < 0.01). Overall median health services costs in the Survivor Cohort were higher among older individuals compared to the younger individuals (age ≥ 65 versus age < 65, *p* < 0.01), but these costs did not significantly differ by age in the Deceased Cohort (*p* = 0.064) (E-Appendix 2).

## 4. Discussion

In this population-based study of 326 treated PAH patients in Ontario, the average drug costs for PAH patients were substantial, exceeding $2,800 per month for those on single therapy and $4,500 per month for those on combination therapy. In addition to high PAH drug costs, we found that the mean costs of health services were substantial for PAH patients, exceeding $750 per month for those who survived for one year and $4,600 per month for those who died during the 1-year follow-up. These costs were 1.5 and 10 times higher than the per-capita health services utilization costs reported by CIHI for the general Ontarian population ($5,835 per year, or approximately $486 per month) [29], highlighting the substantial complexity of care for PAH patients.

In the REVEAL study [5], patients had similar age and gender profiles (mean age = 53, 79.5% female) to our study (mean age = 59, 77% female). This is unlike previous registries, which typically had an approximately 2 : 1 female to male ratio and an older age profile. This perhaps suggests a shift towards greater female preponderance and overall improved survival with evolving therapy. We found PAH drug usage patterns to be largely similar across the registries. However, the REVEAL study in the US had a higher proportion of combination therapy (65%) compared to our study (23%). This may reflect a relatively more restricted access to combination therapy in Ontario due to limited funding by the public drug program. If restricted access to combination therapy in Canada is the underlying cause to this substantial difference, whether or not this has an impact on quality of care, quality of life, and survival is uncertain and merits further investigation.

Follow-up outcomes showed that our overall one-year survival rate (89.6%) was similar to previous contemporary reports in which one-, three-, five-, and seven-year survival were found to be 85%, 68%, 57%, and 49%, respectively [30]. Comparing the Survivor and Deceased Cohorts revealed that patients at end-stage PAH, in their final year of life, had substantially high health services costs compared to individuals who lived for at least one year from the start of follow-up. We also found that PDE5is were least costly because of lower daily drug acquisition costs. Further, there are generally fewer laboratory and diagnostics procedures associated with managing patients on PDE5s [16]. Elsewhere, a population-based cost-minimalization analysis in Canada revealed that overall costs including health services were approximately $48 352 CAD/3-year period for PDE5is and $148 443–$164 745/3-year period for ERAs [16]. Formal cost-effectiveness analyses were out of the scope of our study, but the lack of pharmacoeconomic research in PAH merits further investigation in this area.

A key strength of our study is the population-based nature of the analyses. Although registries provide valuable epidemiological insight, they are imperfect because patients with severe or rapidly progressive disease may not live long enough to be enrolled in the registry due to premature death, while patients with stable disease over a number of years may be overrepresented. Our use of a cross-sectional, population-based analysis allowed us to capture all patients alive on our start date, who had received any treatment for PAH in the prior year. Further, we are confident about the treatment patterns and health services utilization and costs associated with PAH in this patient population. This provides important insight about costs of the disease to payers in an era where novel therapeutic strategies are being investigated to improve survival, reduce hospitalization, and offer some benefit to payers.

However, several limitations of our study merit emphasis. Firstly, we were only able to retrieve data on people with publicly funded PAH treatment. Therefore we are unable to reliably estimate the prevalence of treated PAH using the available data. Secondly, we found that 25 out of 326 identified PAH patients did not have a subsequent PAH prescription over the follow-up period, despite having filled a past PAH prescription. This may be due to misdiagnosis, or the patient moving outside of Ontario, switching to private insurance, or discontinuing treatment. Other potential sources of errors in our method for patient identification may arise from excluding individuals diagnosed but not treated or including those who were overdiagnosed with PAH and treated “off label.” The influences of these factors are likely small as PAH prescriptions are tightly regulated and almost exclusively prescribed through Centres of Excellence. Further, in Ontario, prescribing physicians are generally required to provide hemodynamic data to the ministry as well as assurances for ruling out other relevant burdens of lung disease as a cause for the PH. These include pulmonary embolism and left-sided heart disease, or those with normal pulmonary capillary wedge pressure. Further, although some patients in this era may have already been on PAH drugs and not seen at an expert centre, this is likely very uncommon.

Our high rate of comorbid heart failure likely relates to the fact that PAH patients are often coded to have righted-sided heart failure in administrative database. Finally, although our methods used to calculate costs are employed regularly in health services research [31–33], some limitations merit disclosure. For PROs drug costs, ODB covers the costs of PROs and diluents only. The costs for peripherals, such as pump rental, cassettes, syringes, sterile materials, and needles, are not reflected in our ODB-reported costs. These expenses are covered by the community care access program and we did not account for these costs in our cost analysis. Our cost analysis did not include expenditures related to the operation of the healthcare system. It also excluded the capital costs for large-scale projects, such as community-level health services and services where health card numbers are not tracked. Accordingly, the costs for these services are not reflected in our person level cost estimates. Marginal cost analyses and incremental cost analyses were also excluded. Costs associated with specific technologies such as CT and MRI scans, performed within acute care hospitals, were also excluded.

## 5. Conclusion

With targeted therapies, PAH patients have a higher life expectancy and better quality of life than a few decades ago [34]. This has improved the prognosis of PAH by delaying disease progression [35]. However, survival is still low and we need improved surveillance and new medical therapies for better patient management. This study describes characteristic of treated PAH patients and highlights the high health services utilization and costs associated with PAH in Ontario. This emphasizes the need to develop appropriate PAH treatment strategies in this group of complex patients. Further basic, clinical, and surveillance research may help guide towards developing appropriate and timely treatment strategies.

## Supplementary Material

E-Appendix 1 describes the average overall costs of prescription PAH drug therapy in Ontario from April 1, 2011 to March 31, 2012. The costs are reported as per patient per month. The costs of both single drug therapy as well as combination drug therapy for PAH are described. The data are also stratified by age.
E-Appendix 2 describes the average health services utilization and associated average costs for individuals treated with a PAH drug in Ontario from April 1, 2011 to March 31, 2012. The data describe health services utilization and costs for physician visits, hospitalizations, and emergency department visits. The data are stratified into two cohorts: those who survived a one-year follow-up period and those who did not survive a one-year follow-up period. The data are also stratified by age.

## Figures and Tables

**Table 1:** Baseline characteristics and demographics of PAH cases in Ontario as of April 1, 2011.

	Total	Age < 65	Age ≥ 65	*p* value Age < 65 versus age ≥ 65
	*N* = 326	*N* = 165	*N* = 161	
Female (*N* (%))	251 (77%)	127 (77%)	124 (77%)	0.992
Age (mean, SD)	59.4 (20.3)	43.4 (15.8)	75.7 (6.7)	<0.001
Rural location of residence (*N* (%))	39 (12%)	17 (10.3%)	22 (13.7%)	0.350
Income quintile (*N* (%))				
1 (lowest)	77 (23.6%)	42 (25.5%)	35 (21.7%)	0.430
2	52 (16%)	29 (17.6%)	23 (14.3%)	0.417
3	64 (19.6%)	31 (18.8%)	33 (20.5%)	0.698
4	81 (24.9%)	37 (22.4%)	44 (27.3%)	0.306
5 (highest)	52 (16%)	26 (15.8%)	26 (16.2%)	0.923
Comorbidities (*N* (%))				
Cardiovascular disease	312 (95.7%)	156 (94.6%)	156 (96.9%)	0.296
Heart failure	72 (22.1%)	33 (20%)	39 (24.2%)	0.358
Atrial fibrillation/flutter	41 (12.6%)	9 (5.5%)	32 (19.9%)	0.001
Respiratory disease	154 (47.2%)	56 (33.9%)	98 (60.9%)	<0.001
Chronic obstructive pulmonary disease	147 (45.1%)	55 (33.3%)	92 (57.1%)	<0.001
Connective tissue disease	47 (14.4%)	27 (16.4%)	20 (12.4%)	0.311
Other conditions				
Diabetes	79 (24.2%)	27 (16.4%)	52 (32.3%)	<0.001
Thyroid disease	17 (5.2%)	10 (6.1%)	7 (4.4%)	0.487
Drug use (past 3 years) (*N* (%))				
Antihypertensive	145 (44.5%)	49 (29.7%)	96 (59.6%)	<0.001
Calcium channel blockers	75 (23%)	19 (11.5%)	56 (34.8%)	<0.001
Oral anticoagulants	125 (38.3%)	53 (32.1%)	72 (44.7%)	0.019
Diuretics	189 (58.0%)	73 (44.2%)	116 (72.1%)	<0.001
Digoxin	25 (7.7%)	9 (5.5%)	16 (9.9%)	0.128

**Table 2:** Overall and age-stratified PAH drug therapy utilization patterns in Ontario between April 1, 2011, and March 31, 2012.

PAH drug therapy	Overall *N* = 301^*∗*^	Age < 65 *N* = 152	Age ≥ 65 *N* = 149	*p* value Age < 65 versus age ≥ 65
	*N* (%)	*N* (%)	*N* (%)	
Overall single therapy	232 (77.1%)	118 (77.6%)	114 (76.5%)	0.817
Overall combination therapy	69 (22.9%)	34 (22.4%)	35 (23.5%)	0.817
Types of single therapy				
PDE5 inhibitors	66 (28.4%)	23–28 (19–24%)^*∗*^	40–45 (35–39%)	0.013
ERA	140 (60.3%)	65–70 (55–59%)	70–75 (61–66%)	0.389
Prostanoids	26 (11.2%)	21–26 (20–22%)	≤5 (0–4%)	<0.001
Types of combination therapy				
PDE5 inhibitors + ERA	62 (89.9%)	28–33 (82–97%)	29–34 (83–97%)	0.661

^*∗*^Ranges provided for privacy reasons to avoid reidentification of small cell sizes.

Note: 25 out of 326 patients did not have a subsequent PAH prescription over the follow-up period, despite having a past PAH prescription, and therefore are excluded from this analysis.

**Table 3:** Average health service utilization costs per month among individuals receiving PAH therapy in the Survivor Cohort and the Deceased Cohort, in Ontario, from April 1, 2011, to March 31, 2012.

	Survivor Cohort	Deceased Cohort	*p* value
	Overall (*N* = 292)	Overall (*N* = 34)	
Overall cost for health services utilization			
Median (IQR)	$264 ($96–$747)	$2,021 ($993–$6,399)	<0.001
Mean (SD)	$751 ($1,461)	$4,669 ($6,551)	<0.001
Physician visits			
Number with any physician visits (*N* (%))	290 (99.3%)	34 (100%)	0.628
Number of physician visits (median (IQR))	28 (17–46)	31.5 (16–46)	0.886
Costs of physician visits (median (IQR))	$160 ($87–$275)	$551 ($301–$769)	<0.001
Hospitalizations			
Number with any hospitalizations (*N* (%))	144 (49.3%)	28 (82.4%)	<0.001
Number of hospitalizations (median (IQR))	1.5 (1-2)	1 (1-2)	0.766
Costs of hospitalizations (median (IQR))	$405 ($152–$889)	$1,820 ($767–$6,332)	<0.001
Emergency department visits			
Number with any emergency department visits (*N* (%))	144 (49.3%)	31 (91.2%)	<0.001
Number of emergency department visits (median (IQR))	2 (1–4)	2 (1–3)	0.922
Costs of emergency department visits (median (IQR))	$62 ($26–$119)	$213 ($101–$392)	<0.001

## Abbreviations

CIHI-DAD: Canadian Institute for Health Information Discharge Abstract Database
CIHI-NACRS: CIHI National Ambulatory Care Reporting System
ERA: Endothelin receptor antagonists
ICD-10: International Classification of Diseases, 10th Revision
ODB: Ontario Drug Benefit
PAH: Pulmonary arterial hypertension
PDE5is: Phosphodiesterase-5 inhibitors
PRO: Prostacyclin.

## Additional Points

This is the first study to describe the characteristics of treated PAH patients in Ontario, as well as their health services utilization and associated costs, using population-based data. This research has not been presented or published elsewhere.

## References

[1] Brown L. M., Chen H., Halpern S. (2011). Delay in recognition of pulmonary arterial hypertension: factors identified from the REVEAL registry. *Chest*.

[2] Gatzoulis M. A. (2012). *Pulmonary Arterial Hypertension*.

[3] Rich S., Dantzker D. R., Ayres S. M. (1987). Primary pulmonary hypertension. A national prospective study. *Annals of Internal Medicine*.

[4] Thenappan T., Shah S. J., Rich S., Gomberg-Maitland M. (2007). A USA-based registry for pulmonary arterial hypertension: 1982–2006. *European Respiratory Journal*.

[5] Badesch D. B., Raskob G. E., Elliott C. G. (2010). Pulmonary arterial hypertension: baseline characteristics from the REVEAL registry. *Chest*.

[6] Kane G. C., Maradit-Kremers H., Slusser J. P., Scott C. G., Frantz R. P., McGoon M. D. (2011). Integration of clinical and hemodynamic parameters in the prediction of long-term survival in patients with pulmonary arterial hypertension. *Chest*.

[7] Peacock A. J., Murphy N. F., McMurray J. J. V., Caballero L., Stewart S. (2007). An epidemiological study of pulmonary arterial hypertension. *European Respiratory Journal*.

[8] Escribano-Subias P., Blanco I., López-Meseguer M. (2012). Survival in pulmonary hypertension in Spain: insights from the Spanish registry. *European Respiratory Journal*.

[9] Ling Y., Johnson M. K., Kiely D. G. (2012). Changing demographics, epidemiology, and survival of incident pulmonary arterial hypertension: results from the pulmonary hypertension registry of the United Kingdom and Ireland. *American Journal of Respiratory and Critical Care Medicine*.

[10] Humbert M., Sitbon O., Chaouat A. (2006). Pulmonary arterial hypertension in France—results from a national registry. *American Journal of Respiratory and Critical Care Medicine*.

[11] Zhang R., Dai L.-Z., Xie W.-P. (2011). Survival of Chinese patients with pulmonary arterial hypertension in the modern treatment era. *Chest*.

[12] Jing Z.-C., Xu X.-Q., Han Z.-Y. (2007). Registry and survival study in Chinese patients with idiopathic and familial pulmonary arterial hypertension. *Chest*.

[13] Hoeper M. M., Huscher D., Ghofrani H. A. (2013). Elderly patients diagnosed with idiopathic pulmonary arterial hypertension: results from the COMPERA registry. *International Journal of Cardiology*.

[14] Pope J. E., Lee P., Baron M. (2005). Prevalence of elevated pulmonary arterial pressures measured by echocardiography in a multicenter study of patients with systemic sclerosis. *Journal of Rheumatology*.

[15] Shimony A., Fox B. D., Langleben D., Rudski L. G. (2013). Incidence and significance of pericardial effusion in patients with pulmonary arterial hypertension. *Canadian Journal of Cardiology*.

[16] Dranitsaris G., Mehta S. (2009). Oral therapies for the treatment of pulmonary arterial hypertension: a population-based cost-minimization analysis. *Applied Health Economics and Health Policy*.

[17] Iskedjian M., Walker J., McLean A., Farah B., Berbari J., Watson J. (2012). PRS20 cost-benefit analysis for a treatment of pulmonary arterial hypertension. *Value in Health*.

[18] Einarson T. R., Granton J. T., Vicente C., Walker J. H., Engel G., Iskedjian M. (2005). Cost-effectiveness of treprostinil versus epoprostenol in patients with pulmonary arterial hypertension: a Canadian analysis. *Canadian Respiratory Journal*.

[19] Angalakuditi M., Edgell E., Beardsworth A., Buysman E., Bancroft T. (2010). Treatment patterns and resource utilization and costs among patients with pulmonary arterial hypertension in the United States. *Journal of Medical Economics*.

[20] Wilkens H., Grimminger F., Hoeper M. (2010). Burden of pulmonary arterial hypertension in Germany. *Respiratory Medicine*.

[21] Galiè N., Manes A., Negro L., Palazzini M., Bacchi-Reggiani M. L., Branzi A. (2009). A meta-analysis of randomized controlled trials in pulmonary arterial hypertension. *European Heart Journal*.

[22] Humbert M., Sitbon O., Simonneau G. (2004). Treatment of pulmonary arterial hypertension. *The New England Journal of Medicine*.

[23] Borrie A. E., Ostrow D. N., Levy R. D., Swiston J. R. (2011). Assessing response to therapy in idiopathic pulmonary arterial hypertension: a consensus survey of Canadian pulmonary hypertension physicians. *Canadian Respiratory Journal*.

[24] Galie N., Hoeper M. M., Humbert M. (2009). Guidelines for the diagnosis and treatment of pulmonary hypertension: the Task Force for the Diagnosis and Treatment of Pulmonary Hypertension of the European Society of Cardiology (ESC) and the European Respiratory Society (ERS), endorsed by the International Society of Heart and Lung Transplantation (ISHLT). *European Heart Journal*.

[25] Barst R. J., Gibbs J. S. R., Ghofrani H. A. (2009). Updated evidence-based treatment algorithm in pulmonary arterial hypertension. *Journal of the American College of Cardiology*.

[26] National Pulmonary Hypertension Centres of the UK and Ireland (2008). Consensus statement on the management of pulmonary hypertension in clinical practice in the UK and Ireland. *Heart*.

[27] Simonneau G., Gatzoulis M. A., Adatia I. (2013). Updated clinical classification of pulmonary hypertension. *Journal of the American College of Cardiology*.

[28] Wodchis W. P. B., Nikitovic M., McKillop I. (2013). Guidelines on person-level costing using administrative databases in Ontario. *Network HSPR*.

[29] Canadian Institute for Health Information (2013). *National Health Expenditure Trends, 1975 to 2013*.

[30] Benza R. L., Miller D. P., Barst R. J., Badesch D. B., Frost A. E., McGoon M. D. (2012). An evaluation of long-term survival from time of diagnosis in pulmonary arterial hypertension from the REVEAL registry. *Chest*.

[31] Gomes T., Mamdani M. M., Holbrook A. M., Paterson J. M., Hellings C., Juurlink D. N. (2013). Rates of hemorrhage during warfarin therapy for atrial fibrillation. *Canadian Medical Association journal*.

[32] Gomes T., Juurlink D. N., Dhalla I. A., Mailis-Gagnon A., Paterson J. M., Mamdani M. M. (2011). Trends in opioid use and dosing among socio-economically disadvantaged patients. *Open Medicine*.

[33] Gomes T., Juurlink D. N., Shah B. R., Hellings C. R., Paterson J. M., Mamdani M. M. (2013). Progression through diabetes therapies among new elderly users of metformin: a population-based study. *Diabetic Medicine*.

[34] Mehta S., Shoemaker G. J. (2005). Improving survival in idiopathic pulmonary arterial hypertension: revisiting the ‘kingdom of the near-dead’. *Thorax*.

[35] Rosanio S., Pelliccia F., Gaudio C., Greco C., Keylani A. M., D'Agostino D. C. (2014). Pulmonary arterial hypertension in adults: novel drugs and catheter ablation techniques show promise? Systematic review on pharmacotherapy and interventional strategies. *BioMed Research International*.

