# Supplementary material for: The Characteristics of Treated Pulmonary Arterial Hypertension Patients in Ontario
Source: Can Respir J. 2016 Apr 11;2016:6279250. doi: 10.1155/2016/6279250 (PMC4904539; doi:10.1155/2016/6279250)
Supplement: Supplementary file 1 — E-Appendix 1 describes the average overall costs of prescription PAH drug therapy in Ontario from April 1, 2011 to March 31, 2012. The costs are reported as per patient per month. The costs of both single drug therapy as well as combination drug therapy for PAH are described. The data are also stratified by age. E-Appendix 2 describes the average health services utilization and associated average costs for individuals treated with a PAH drug in Ontario from April 1, 2011 to March 31, 2012. The data describe health services utilization and costs for physician visits, hospitalizations, and emergency department visits. The data are stratified into two cohorts: those who survived a one-year follow-up period and those who did not survive a one-year follow-up period. The data are also stratified by age. [file 6279250.f1.pdf]

E-Appendix 1: Average Overall, and Age-Stratified, Single and Combination Prescription PAH Drug Therapy Costs per Month in Ontario, from April 1, 2011 to March 31, 2012

PAH Drug Therapy	Overall (N=301) Average Costs per Month (Mean, SD)	Aged<65 (N=152) Average Costs per Month (Mean, SD)	Aged>=65 (N=149) Average Costs per Month (Mean, SD	p-value Age<65 vs. Age ≥ 65
Overall Single Therapy	$2,801 ($1,550)	$2,833 ($1,546)	$2,767 ($1,561)	0.746
Overall Combination Therapy	$4,569 ($1,544)	$4,420 ($1,504)	$4,713 ($1,591)	0.435
TYPES OF SINGLE THERAPY INITIATED				
PDE5 Inhibitors	$880 ($380)	$816 ($410)	$920 ($359)	0.288
ERA	$3,609 ($1,043)	$3,447 ($1,256)	$3,762 ($768)	0.074
Prostanoids	$3,324 ($1,451)	$3,182 ($1,282)	—*	-
TYPES OF COMBINATION THERAPY				
PDE5 Inhibitors + ERA	$4,298 ($917)	$4,264 ($970)	$4,331 ($879)	0.777
All Others	$6,964 ($3,322)	$5,592 ($3,701)	$8,793 ($1,942)	0.237
All patients on PAH Therapy	$3,206 ($1,716)	$3,188 ($1,669)	$3,224 ($1,768)	0.856

*Suppressed to protect privacy since ≤5 individuals are present within this patient group.

E-Appendix 2: Average Age-Stratified Health Services Utilization and Costs for the Survivor Cohort and the Deceased Cohort in Ontario, from April 1, 2011 to March 31, 2012

	Survivor Cohort			Deceased Cohort		
	Age<65 (N=155)	Age ≥ 65 (N=137)	p-value<br>Age<65 vs. Age ≥ 65	Age<65 (N=10)	Age ≥ 65 (N=24)	p-value<br>Age<65 vs. Age ≥ 65
OVERALL COST FOR ALL HEALTH SERVICES UTILIZATION<br>Median (IQR)	$172 ($79 -$587)	$357 ($136-$919)	<.001	$4,139 ($1,817-$11,611)	$1,782 ($823-$3,205)	0.064
PHYSICIAN VISITS						
Number with any Physician Visits [N (%)]	154 (99.4%)	136 (99.3%)	0.93	10 (100%)	24 (100%)	n/a
Number of Physician Visits<br>[Median (IQR)]	21.5 (14-39)	35 (23-51)	<.001	17.5 (15.0-34.0)	36 (16.5-47)	0.354
Costs of Physician Visits<br>[Median (IQR)]	$130 ($73-$221)	$195 ($113-$289)	<.001	$661 ($206-$1,588)	$493 ($307-$709)	0.29
HOSPITALIZATIONS						
Number with any Hospitalizations [N (%)]	63 (40.6%)	81 (59.1%)	0.002	9 (90.0%)	19 (79.2%)	0.45

Number of Hospitalizations<br><br>[Median (IQR)]	1 (1-2)	2 (1-2)	0.849	2 (1-2)	1 (1-2)	0.203
Costs of Hospitalizations<br><br>[Median (IQR)]	$364 ($156-$843)	$436 ($136-$903)	0.828	$3,419 ($1,576-$8,597)	$1,304 ($645-$4,960)	0.147
EMERGENCY DEPARTMENT VISITS						
Number with any Emergency Department Visits [N (%)]	70 (45.2%)	74 (54%)	0.131	10 (100%)	21 (87.5%)	0.242
Number of Emergency Department Visits<br><br>[Median (IQR)]	2 (1-4)	2 (1-4)	0.887	2 (1-3)	2 (1-3)	0.641
Costs of Emergency Department Visits<br><br>[Median (IQR)]	$57 ($20-$114)	$65 ($32-$126)	0.167	$250 ($167-$455)	$193 ($101-$292)	0.331
